# 1025. Case Study. Folliculitis and Pseudofolliculitis Barbae in Burn Survivors: A Clinically Significant yet Underrecognized and Understudied Challenge

**DOI:** 10.1093/jbcr/irag033.524

**Published:** 2026-04-06

**Authors:** Taryn Dee, Amber D Kohler, Cameron Gibson, Alexandra Halevi, Fred Endorf, Arek J Wiktor

**Affiliations:** University of Colorado / UCHealth Burn & Frostbite Center, Aurora, Colorado; University of Colorado / UCHealth Burn & Frostbite Center, Aurora, Colorado; University of Colorado, Aurora, Colorado; University of Colorado School of Medicine, Aurora, Colorado; University of Colorado School of Medicine, Aurora, Colorado; University of Colorado / UCHealth Burn & Frostbite Center, Aurora, Colorado

## Abstract

**Patient Presentation (age range, injury details, relevant history):**

There were five patients who presented to our ABA certified burn center in 2024 who later developed signs and symptoms of folliculitis barbae (FB) or pseudofolliculitis barbae (PFB). All were male with an average age of 44 years (range 36-50) and average TBSA of 28.22% (range 16.25%-49.25%). Four of the five sustained burns from flame, the fifth from a flash burn. All sustained partial- to full-thickness facial and neck burns with two requiring intubation. All five patients were shaved regularly and underwent daily wound care. None received facial skin grafting for the initial burn injury. Fitzpatrick skin types ranged from II to IV, with three of the five patients identifying as white, one as native and one African American. Three patients had positive yeast and Staph lugdunensis cultures taken from affected areas.

**Clinical Challenges:**

FB is an underreported complication in burn patients, primarily affecting individuals with superficial to deep partial-thickness burns to hair-bearing areas. To date, there are no published studies addressing PFB in burn survivors. Additionally, there are no established diagnostic criteria, severity grading scales, or standardized treatment protocols for FB or PFB in this population.

**Management Approach:**

All five patients were treated with topical mupiricon, and three were prescribed triamcinolone ointment in conjunction. One patient was treated with topical antifungals, and another patient received a short course of oral doxycycline. Four of the five patients were treated with a course of laser therapy and triamcinolone acetonide injections. Three were referred to dermatology for hair removal treatment. One received reconstruction with full thickness excision and skin grafting.

**Outcomes:**

Two patients responded well to a combination of topical antibiotics, laser therapy and triamcinolone acetonide injections. Despite the same treatment one patient required deep excision and skin grafting. Another patient’s symptoms resolved with referral to dermatology. The final patient was lost to follow up.

**Lessons Learned:**

FB and PFB represents a clinically relevant but underrecognized complication in particularly men with facial and neck burns. The wide variability in presentation, time to development, and treatment response highlights the need for greater awareness, along with research and development of standardized diagnostic criteria and treatment protocols.

**Applicability to Practice:**

The delayed time to symptoms development suggests that this complication may emerge well after the acute recovery phase, possibly as hair regrows in the area. When treating FB and PFB burn centers should consider a multidisciplinary approach with dermatology. Biopsy and culture results may also help guide treatment.

